# Supplementary material for: Esophageal Cancer Stem-like Cells Resist Ferroptosis-Induced Cell Death by Active Hsp27-GPX4 Pathway
Source: Biomolecules. 2021 Dec 29;12(1):48. doi: 10.3390/biom12010048 (PMC8773805; doi:10.3390/biom12010048)
Supplement: Supplementary file 1 [file biomolecules-12-00048-s001.zip › biomolecules-1517416-supplementary.pdf]

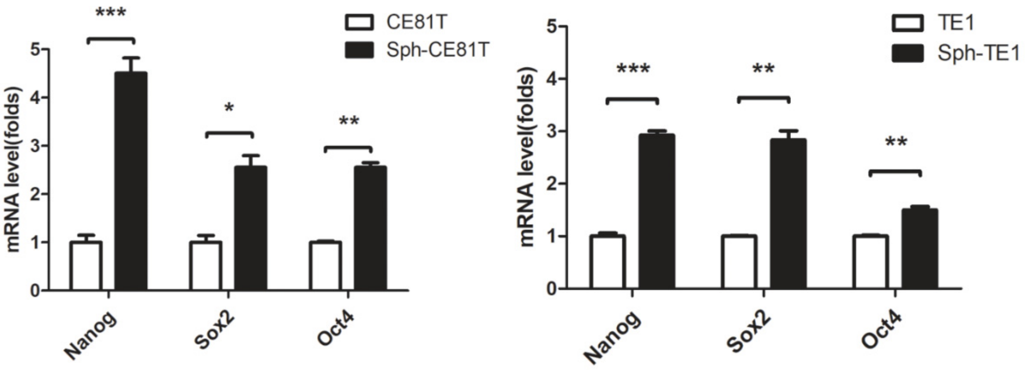

Supplementary Figure S1. Real-time PCR of Nanog, Sox2, and Oct4 of CE81T and TE1 cells in spheroid (Sph) and adherent cultures.

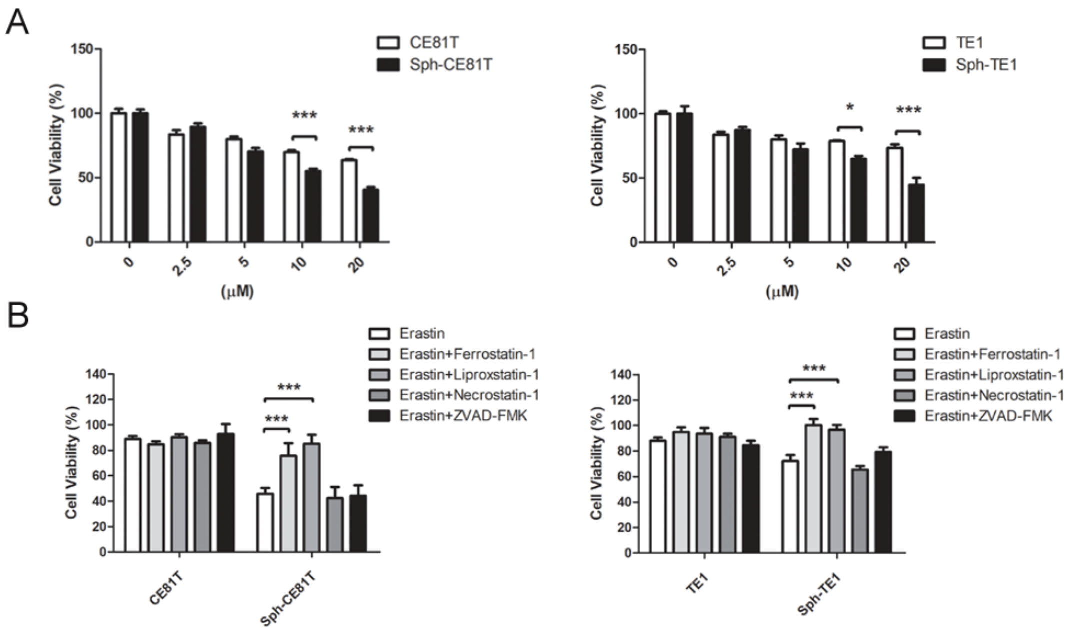

Supplementary Figure S2. Erastin induced more ferroptosis in esophageal cancer cells in spheroid culture.

(A) Cell viability of TE1 and CE81T cells cultured in spheroid (Sph-TE1, Sph-CE81T) or adherent system and treated with erastin in different concentration to induce ferroptosis. (B) Viability of cells in different cultures with erastin (20 μM) were treated with ferrostatin-1 (1 μM), liproxstatin-1 (1 μM), ZVAD-FMK (50 μM), or Necrostatin-1 (50 μM) for 24 h.

Supplementary Table S1. Demographic data of 70 patients who underwent surgery for esophageal cancer.

Variables (n=70)	Number(%)
Age(year)	38-91(69.4±10.5)
Sex	
Male	67(95.7)
Female	3(4.3)
Histological type	
Epidermoid carcinoma	16(22.9)
Squamous cell carcinoma	52(74.3)
Adenosqumous carcinoma	1(1.4)
Epidermoid carcinoma, adenocarcinoma	1(1.4)
T status	
T1	4(5.7)
T2	13(18.6)
T3	45(64.3)
T4	8(11.4)
N status	
N0	31(44.3)
N1	37(52.9)
N2	2(2.9)
M status	
M0	62(88.6)
M1	8(11.4)
pHsp27 expression	
Increased expression (+)	16(22.9)
Decreased expression (-)	54(77.1)
GPX4 expression	
Increased expression (+)	55(78.6)
Decreased expression (-)	15(21.4)
pHsp27 and GPX4 coexpression	
pHsp27 (+)/GPX4 (+)	14(20)
pHsp27 (+)/GPX4 (-)	2(2.9)
pHsp27 (-)/GPX4 (+)	41(58.6)
pHsp27 (-)/GPX4 (-)	13(18.6)
